# Indications for cesarean delivery among patients with life‐limiting fetal diagnoses

**DOI:** 10.1002/pmf2.70429

**Published:** 2026-08-21

**Authors:** Halle C. Petrie, Alexandra C. Sundermann, B. Wade Hamilton, Allison M. McCarthy, Emily A. Morris, Luke A. Gatta

**Affiliations:** ^1^ Vanderbilt University School of Medicine Nashville Tennessee USA; ^2^ Division of Quantitative and Clinical Sciences Vanderbilt Health Nashville Tennessee USA; ^3^ Division of Psychiatry and Behavioral Sciences Vanderbilt Health Nashville Tennessee USA; ^4^ Center for Biomedical Ethics and Society Vanderbilt Health Nashville Tennessee USA; ^5^ Division of Neonatology Department of Pediatrics Vanderbilt Health Nashville Tennessee USA; ^6^ Division of Maternal‐Fetal Medicine Department of Obstetrics and Gynecology Vanderbilt Health Nashville Tennessee USA

**Keywords:** abortion, cesarean delivery, fatal fetal diagnosis, Lethal, life‐limiting diagnosis, pediatric hospice, pediatric palliative care

## Abstract

**Introduction:**

Mode of delivery is uniquely complex when a neonate is not expected to survive after birth. Cesarean delivery (CD) generally confers increased maternal morbidity but may be pursued to mitigate intrapartum fetal risk. Prior studies have reported elevated CD rates among patients with a life‐limiting fetal diagnosis (LLFD) but have not characterized the indications that drive this increase. We sought to characterize the proportion and indications for CD among patients with selected LLFDs.

**Methods:**

This retrospective cohort includes patients with an antenatal LLFD who delivered at a tertiary care center between January 1, 2018 and May 1, 2025 at or above 24 weeks' gestation. LLFD cases were chosen for inclusion after manual review by Maternal‐Fetal Medicine and Neonatology subspecialists, and the cohort was further restricted to six LLFDs: anencephaly, trisomy 13, trisomy 18, anhydramnios due to genitourinary etiologies, limb‐body wall complex, and severe skeletal dysplasia. The primary outcome was CD; the secondary outcome was indication for CD.

**Results:**

Of 32,523 eligible deliveries, 241 were identified as having an antenatal LLFD; after restriction to six prespecified diagnoses, the final LLFD cohort included 106 patients. CD occurred in 42.5% of LLFD pregnancies. Proportion of deliveries by CD varied by diagnosis: 0% in anencephaly, 36.4% in skeletal dysplasia, 40.0% in trisomy 13, 45.8% in trisomy 18, 40.0% in limb/body wall complex, and 54.5% in anhydramnios due to a genitourinary cause. A total of 53.3% of CDs were performed for a fetal indication, most commonly malpresentation and non‐reassuring fetal status.

**Conclusion:**

Driven by fetal indications for delivery, patients with a known LLFD underwent CD in 42.5% of pregnancies, with substantial variation across diagnoses. It is unclear whether the increased proportion of CD is driven by traditional obstetric indications or patient‐centered choices regarding the delivery experience.

## 1 INTRODUCTION

Approximately 2%–3% of pregnancies are affected by a congenital anomaly, of which approximately 10% may be considered life‐limiting [1, 2, 3]. Despite the clinical importance of this designation, there is no standardized definition of “life‐limiting”; terms such as “lethal,” “fatal,” and “incompatible with life” are often used interchangeably, depending on the context [4]. Regardless of chosen terminology, these diagnoses are associated with a high likelihood of stillbirth or neonatal death shortly after birth. Both the Society for Maternal‐Fetal Medicine (SMFM) and the American College of Obstetricians and Gynecologists (ACOG) recommend offering pregnancy termination following diagnosis [3, 5].

In the contemporary legal landscape, access to abortion care may be limited for some patients. Others may elect to continue the pregnancy regardless of legal access for a variety of personal or cultural reasons [6]. When a pregnancy is continued after diagnosis, both SMFM and ACOG emphasize the importance of coordinated, multidisciplinary care and individualized, values‐concordant decision‐making, with particular attention to minimizing suffering and honoring patient goals [7, 8]. Within this framework, delivery planning—and specifically mode of delivery—becomes a central and complex decision, as cesarean and vaginal birth may confer differing profiles of maternal morbidity and intrapartum fetal risk.

Prior data suggest a higher rate of cesarean delivery (CD) among patients with anencephaly and trisomy 18, though the reasons for this remain unclear [9, 10]. Decision‐making for CD in the setting of a life‐limiting fetal diagnosis (LLFD) raises distinct ethical and clinical challenges. For example, CD is associated with increased maternal morbidity, and when neonatal survival is expected to be brief or unlikely, traditional fetal‐centered indications for cesarean—such as non‐reassuring fetal status or malpresentation—may be of limited benefit to anticipated neonatal survival [11, 12]. At the same time, knowing their child's life may be brief, some patients may pursue cesarean to increase the possibility of a live birth to facilitate bonding or other meaningful birth experiences [13]. Accordingly, SMFM guidance prioritizes maternal safety and respect for patient autonomy in determining mode of delivery in this setting [5]. Given these competing considerations, and to understand the higher rate of CD (CD) reported in the literature, we sought to characterize the proportion and clinical indications for CD among patients with an antenatal LLFD.

## 2 MATERIALS AND METHODS

This retrospective cohort study included patients with an antenatal diagnosis of an LLFD who delivered at a single tertiary care center in the southeastern United States between January 1, 2018 (earliest available data) and May 1, 2025. The primary outcome was CD. The secondary outcomes were indications for cesarean assessed within the LLFD cohort.

Patients were included if they received prenatal care and underwent vaginal or CD at ≥24 weeks’ gestation. Patients were excluded if they underwent induction of labor prior to 24 weeks’ gestation. Additionally, pregnancies complicated by antepartum intrauterine fetal demise were excluded, as delivery planning in that setting is less likely to be driven by the presence of an LLFD. Twin gestations were excluded, as decision‐making for delivery would be expected to be influenced by the presence of a non‐anomalous fetus.

An LLFD was defined according to ACOG as a fetal condition associated with little to no chance of survival, profound morbidity, diminished quality of life, and absence of curative treatment [3].

Given the absence of a standardized list and the inherent subjectivity of LLFD classification, we used four independent strategies to screen for the study cohort within the electronic medical record (Epic Systems Corporation, Version 2025). Specifically, we identified: (1) pregnancies with both Maternal‐Fetal Medicine (MFM) and Pediatric Palliative Care consultation; (2) stillbirths at ≥24 weeks’ gestation; (3) neonates who died within 28 days of life; and (4) neonates discharged to hospice within 28 days of life. Next, after removing duplicates, all cases were then independently reviewed for inclusion by a board‐certified MFM specialist and, if needed, a neonatologist to confirm that the underlying congenital diagnosis met the ACOG definition for an LLFD. This multi‐strategy approach was designed to maximize sensitivity for LLFD cohort identification: ICD‐10 coding for these fetal diagnoses is inconsistently applied across prenatal, delivery, and neonatal encounters, and several diagnoses of interest (e.g., renal‐cause anhydramnios) do not map cleanly to a single code. Manual chart review would be expected to allow us to identify participants who may have been missed by a code‐based query alone.

We obtained information about maternal demographics, fetal diagnoses, mode of delivery, and delivery indication using the electronic health record related to prenatal care and delivery hospitalization.

For the secondary outcomes, we categorized the indications for CD as fetal (non‐reassuring fetal status, malpresentation, cord prolapse), combined maternal‐fetal (arrest of dilation, arrest of descent, history of myomectomy or CD, abnormal placenta location, anticipated cephalopelvic disproportion, placental abruption), or elective (if the operative note explicitly stated the surgery was elective).

We additionally examined CD proportions across pre‐defined diagnostic subgroups, including anencephaly, trisomy 13, trisomy 18, second trimester anhydramnios due to genitourinary etiologies (bilateral multicystic dysplastic kidneys, renal agenesis, lower urinary tract obstruction), limb‐body wall complex, and severe skeletal dysplasia.

Continuous maternal demographics are presented as medians and interquartile ranges. Categorical characteristics are presented as counts and proportions. We calculated the proportion of deliveries performed via CD overall, by fetal presentation, and by fetal diagnosis. Among our cohort, fetal viability was confirmed via cardiac activity at the start of delivery, and we further stratified CD proportions by fetal presentation (cephalic vs. non‐cephalic). Analyses were performed using Stata 19.0. All statistical tests were two‐sided with a significance threshold of *p* < 0.05.

This study was approved by the institutional review board (#250859). Statistical code and further analytic methods are available upon request.

## 3 RESULTS

**FIGURE 1:**
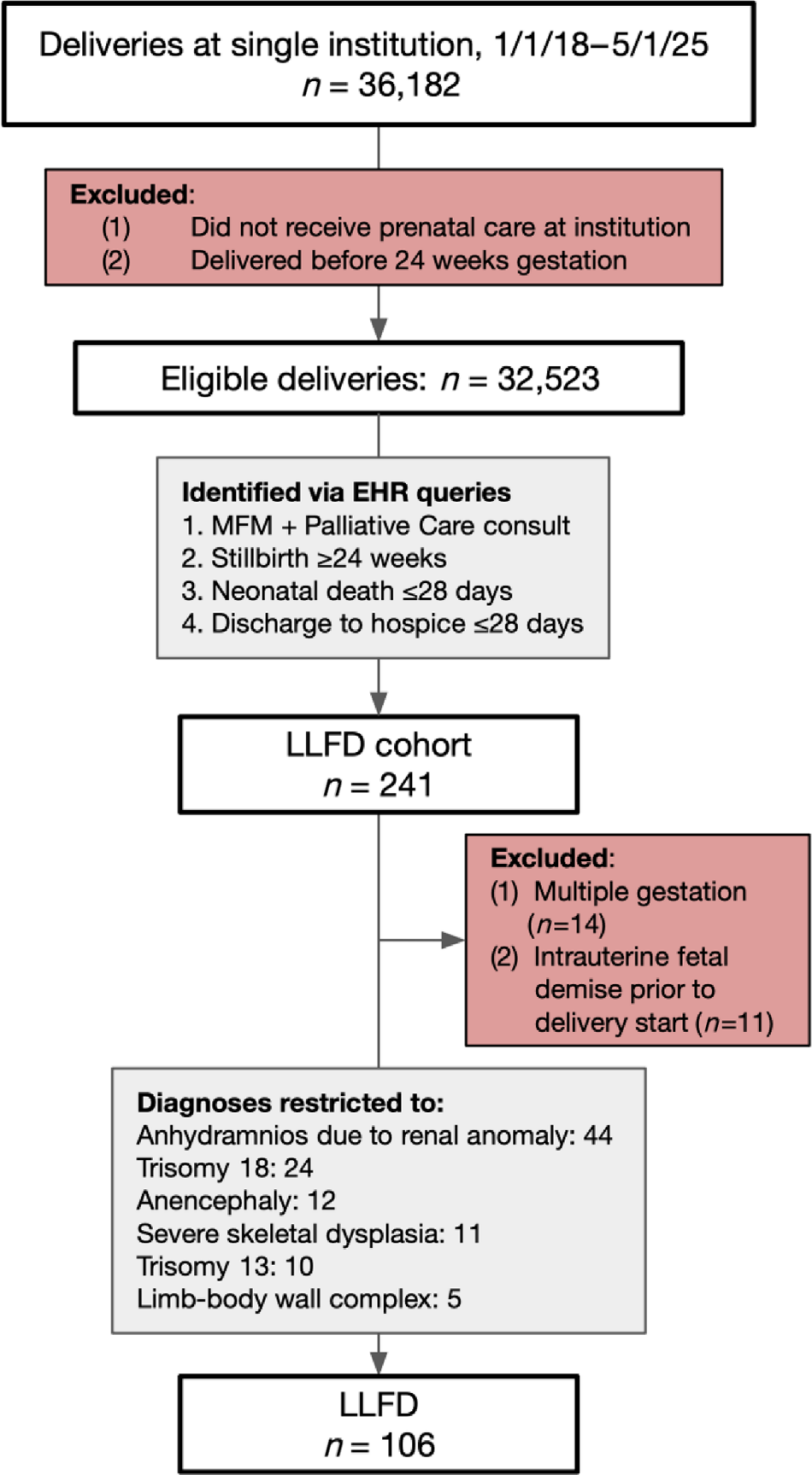
Identification of the study cohort. EHR, electronic health record; LLFD, life‐limiting fetal diagnosis; MFM, Maternal‐Fetal Medicine.

Between January 1, 2018 and May 1, 2025, we identified 241 participants with an antenatal LLFD. After restricting the cohort to the six prespecified diagnoses, the final LLFD cohort included 106 participants: anencephaly (12), trisomy 13 (10), trisomy 18 (24), second trimester anhydramnios due to a genitourinary anomaly (44), severe skeletal dysplasia (11), and limb/body wall complex (5) (Figure 1).

**FIGURE 2:**
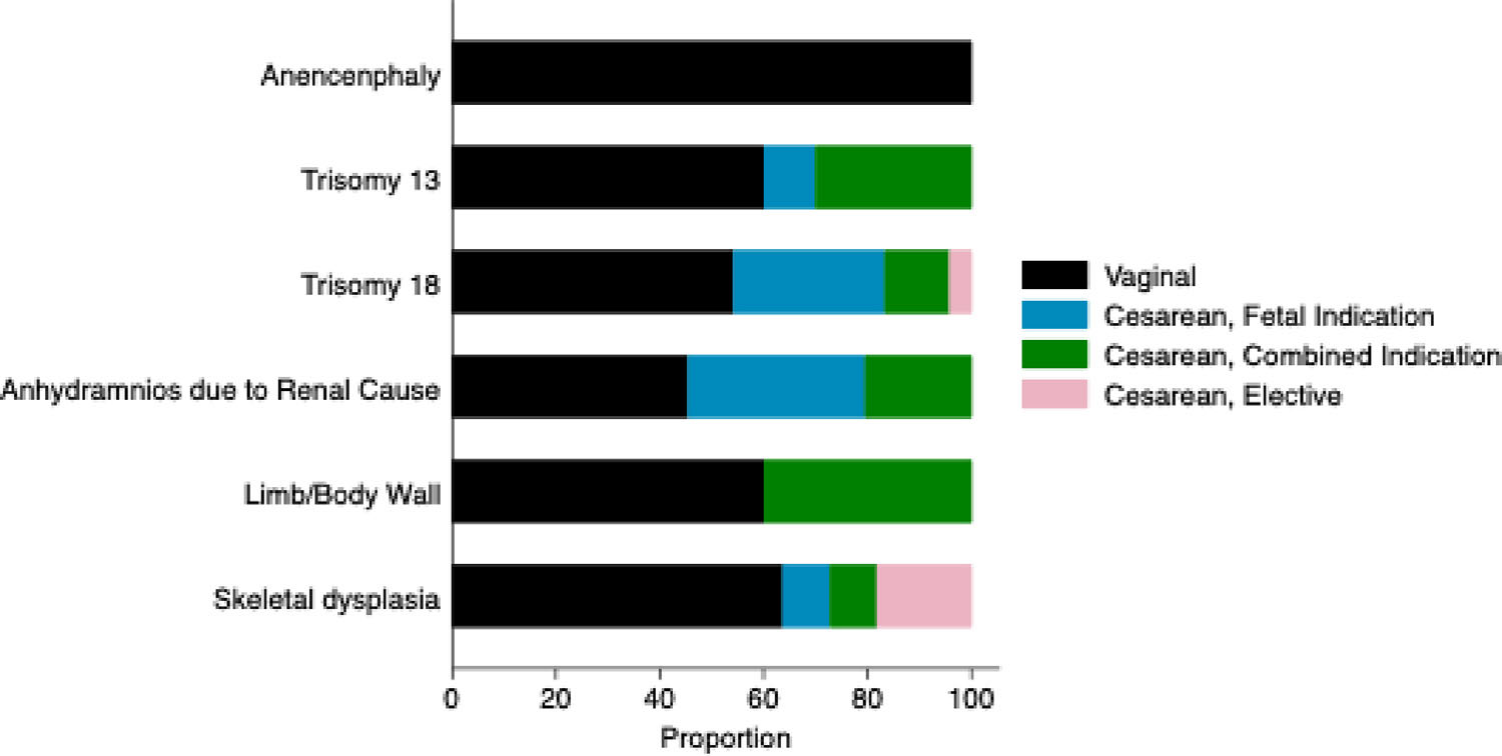
Mode of delivery and cesarean delivery indication among life‐limiting fetal diagnosis pregnancies, stratified by fetal diagnosis (*n* = 106).

Of the 106 LLFD cohort deliveries, 42.5% were via CD. Within the LLFD cohort, delivery patterns varied between fetal diagnoses (Figure 2). No patients with anencephaly (0/12) underwent CD, while CD proportions were 40.0% in trisomy 13 (4/10), 45.8% in trisomy 18 (11/24), 54.5% in second trimester anhydramnios due to a genitourinary cause (24/44), 40.0% in limb/body wall complex (2/5), and 36.4% in skeletal dysplasia (4/11). Baseline maternal characteristics are shown in Table 1. In comparison, the institutional non‐LLFD CD proportion during the study period was 32.7% (among 29,326 deliveries).

**TABLE 1:** Maternal demographic and clinical characteristics.

	LLFD cohort (*n* = 106)
Maternal age at diagnosis Median (IQR)	29 (24, 35)
Gestational age at delivery, weeks Median (IQR)	34 (31, 36)
Gravidity Median (IQR)	2 (1, 4)
Parity Median (IQR)	1 (0, 2)
BMI Median (IQR)	32 (28, 37)
Prior cesarean *n* (%)	17 (16.0%)
Pre‐gestational diabetes *n* (%)	3 (2.8%)
Gestational diabetes *n* (%)	9 (8.5%)

Abbreviations: BMI, body mass index; IQR, interquartile range; LLFD, life‐limiting fetal diagnosis.

**FIGURE 3:**
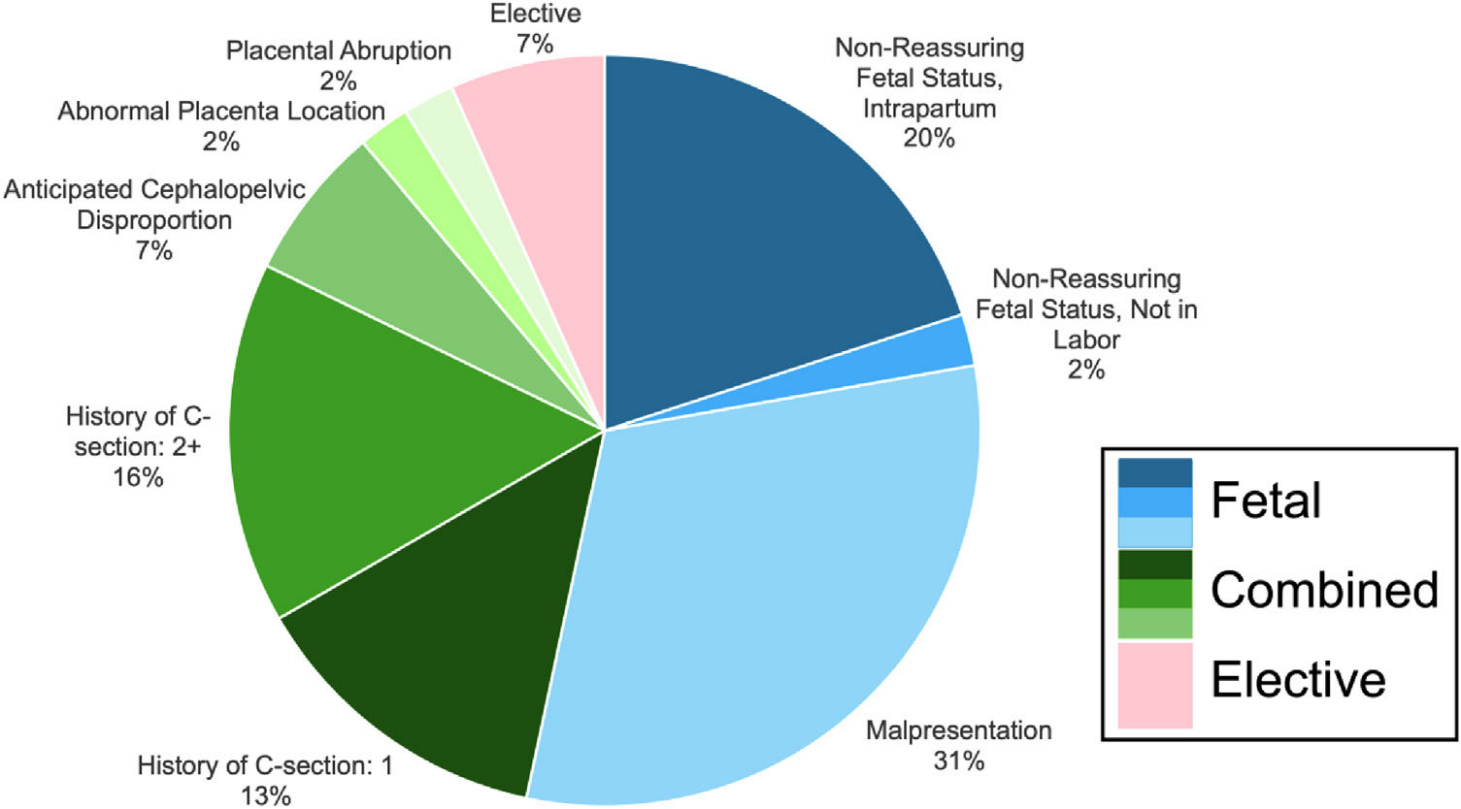
Distribution of indications for cesarean delivery within each category among patients in the life‐limiting fetal diagnosis cohort who underwent cesarean delivery (*n* = 45). Indications are mutually exclusive; percentages reflect the overall proportion of cesarean delivery indications. Fetal indications include non‐reassuring fetal status and malpresentation. Combined maternal‐fetal indications include prior cesarean delivery, anticipated cephalopelvic disproportion, abnormal placental location, and placental abruption.

Of the 45 CDs in the LLFD cohort, over half were performed for a fetal indication (24/45, 53.3%, Table 2; Figure 3). The most common fetal indications were malpresentation (14/45, 31.1%) and non‐reassuring fetal status (10/45, 22.2%, identified either intrapartum or antepartum). Prior CD was the most common combined maternal‐fetal indication (28.9% of all CD). There were no CD for arrest of dilation or arrest of descent in the LLFD cohort. Postpartum hemorrhage occurred in three of the 106 LLFD deliveries (2.8%); all three occurred among patients who underwent CD (3/45, 6.7%).

**TABLE 2:** Indications for cesarean delivery among patients in the LLFD cohort who underwent cesarean delivery (*n* = 45).

Cesarean indication	Cesarean deliveries with LLFD (*n* = 45) *n* (%)
**Fetal Indication**	24 (53.3%)
Non‐Reassuring Fetal Status, Intrapartum	9 (20.0%)
Non‐Reassuring Fetal Status, Not in Labor	1 (2.2%)
Malpresentation	14 (31.1%)
**Combined Maternal‐Fetal Indication**	18 (40.0%)
History of CD: 1	6 (13.3%)
History of CD: 2+	7 (15.6%)
Anticipated Cephalopelvic Disproportion	3 (6.7%)
Abnormal Placenta Location	1 (2.2%)
Placental Abruption	1 (2.2%)
Elective	3 (6.7%)

*Note*: Indications are mutually exclusive and assigned based on the primary documented indication. Fetal indication includes non‐reassuring fetal status, malpresentation, and cord prolapse. Combined maternal‐fetal includes arrest of dilation, arrest of descent, history of myomectomy or cesarean delivery, abnormal placenta location, anticipated cephalopelvic disproportion, and placental abruption.

Abbreviations: CD, cesarean delivery; LLFD, life‐limiting fetal diagnosis.

**FIGURE 4:**
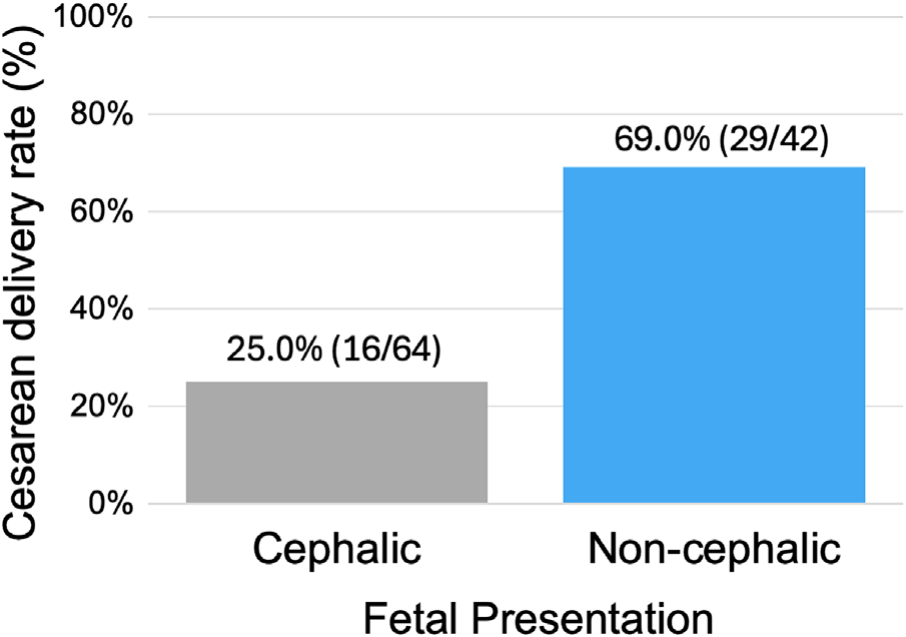
Cesarean delivery proportion among LLFD pregnancies, stratified by fetal presentation (*n* = 106).

The proportion of deliveries via CD differed significantly based on fetal presentation (25.0% CD among those with cephalic fetal presentation [16/64]; 69.0% CD among those with non‐cephalic fetal presentation [29/42], chi‐squared p‐value < 0.01) (Figure 4).

**FIGURE 5:**
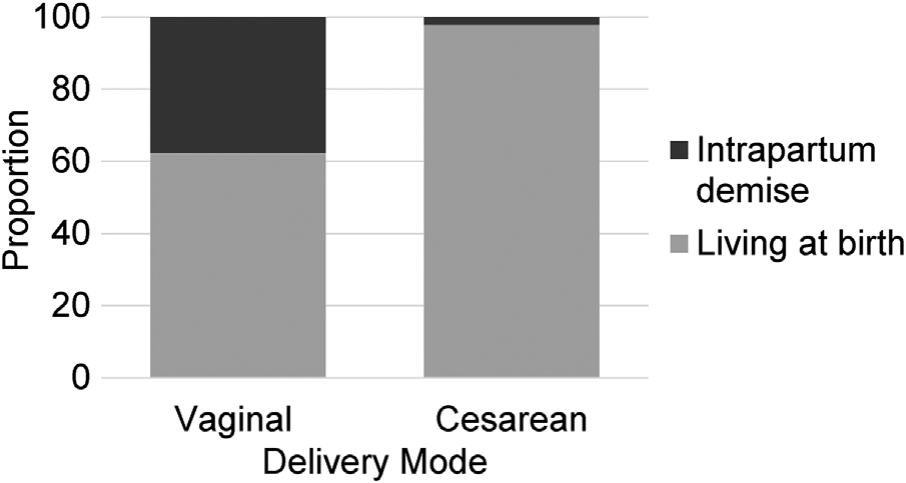
Proportion of live birth and intrapartum demise among patients with a life‐limiting fetal diagnosis (*n* = 106), stratified by mode of delivery.

All 106 pregnancies had a heart rate at the onset of intrapartum course. Intrapartum demise occurred in 22.6% (24/106) of pregnancies overall, with substantial differential by mode of delivery: 37.7% (23/61) among vaginal deliveries versus 2.2% (1/45) among CDs (Figure 5).

## 4 DISCUSSION

In this retrospective cohort study, 42.5% of pregnancies affected by an LLFD delivered via cesarean. Recognizing that local culture impacts CD rate, the institutional CD proportion during the study period was 32.7%. Of the LLFD pregnancies, more than half were driven by a fetal indication, most commonly non‐reassuring fetal status or fetal malpresentation.

This elevated risk for CD patients with an LLFD is consistent with prior studies, both single‐center and national registries [9, 14, 15]. For example, in a single‐center cohort of trisomy 18 pregnancies in a geographically similar region reported a CD rate of 59.3% [10]. A US‐based national birth certificate registry study reported CD rate of 39.2% in anencephalic fetuses versus 31% in non‐anencephalic fetuses [9]. In contrast, in our cohort, no patients with anencephaly underwent CD (0/12), suggesting a patient‐level, institutional, or regional variation in obstetric management of this diagnosis.

The predominance of fetal‐centered indications for cesarean (53.3%) among LLFD pregnancies suggests that mode of delivery may be driven by fetal status—independent of prognosis, with maternal surgical risk incurred largely on behalf of a fetus with limited anticipated survival. Notably, intrapartum demise was substantially more common among LLFD patients who delivered vaginally than among those who underwent cesarean delivery. Because labor and vaginal birth carries inherent physiologic fetal risk, this pattern suggests that avoidance of intrapartum stillbirth was not a universal driver of delivery planning; some patients and clinicians likely accepted this possibility as part of a values‐concordant approach. Consistent with the established maternal morbidity differential between cesarean and vaginal delivery, all three cases of postpartum hemorrhage in our cohort occurred among patients who underwent cesarean; detailed maternal morbidity comparisons fall outside the scope of the present analysis but have been characterized in a recent large LLFD cohort [14]. LLFD pregnancies in our cohort also delivered at a median gestational age of 34 weeks, although it is unclear whether this preterm gestational age reflects intervention for non‐reassuring fetal status during antenatal surveillance, planned early delivery to minimize maternal risk, or a combination of factors.

Two potential explanations may contribute to this pattern of higher cesarean among LLFD pregnancies. First, given the rarity of LLFDs in clinical practice and the absence of clinical management guidelines specifically for LLFD pregnancies, clinicians may default to risk‐benefit frameworks calibrated for anticipated liveborn pregnancies. For instance, in a fetus without anticipated anomalies, a category III fetal heart tracing typically prompts recommendations for immediate cesarean delivery. One might expect that with guarded neonatal survival, such recommendations would weaken in favor of pursuing vaginal birth given the lower associated maternal morbidity, even calling into question the intentions for fetal monitoring itself. Our findings, however, suggest this may not consistently occur. This is particularly notable given prior work in trisomy 18 showing that aggressive obstetric interventions, including CD, do not improve neonatal survival [10]. A second, complementary explanation lies in patient goals. From a strict medical perspective, cesarean for LLFD pregnancies causes harm to the maternal patient without potentially offsetting neonatal benefit. Respect for patients involves recognizing the autonomy of pregnant patients to choose higher personal risks or burdens for the sake of achieving non‐medical benefits [16]. Clinical guidelines from ACOG and SMFM advocate for maternal autonomy as a central focus of antenatal and perinatal care in LLFD pregnancies [3, 5]. Quaresima et al. explicitly weigh maternal well‐being over fetal benefit [12]. Yet, for some families, this means maximizing their chances of meeting a liveborn—as vaginal delivery may pose greater risk for intrapartum demise—is a priority that may render acceptable the surgical risks of cesarean delivery. These nuanced elements of shared decision‐making may not be captured within the medical record, particularly in a retrospective study. Obstetric decision‐making must be responsive to a comfort‐care approach to neonatal end‐of‐life care, including understanding patient goals for their delivery and anticipated brief life of their newborn. Some patients may accept the risk of intrapartum stillbirth, avoiding a cesarean for the indication of fetal benefit when it is not expected to influence survival. Other patients may endeavor to hold a liveborn, even if briefly alive, and therefore accept cesarean if it improves the opportunity to achieve this goal. Intrapartum fetal monitoring becomes an extension of patient goals, developed through shared decision‐making. Several limitations warrant consideration when interpreting our findings. First, as a single‐site retrospective cohort study at a tertiary hospital in the Southeast, our findings may not be generalizable to settings with different patient cultures and legislative contexts. Notably, the CD proportion among our LLFD cohort was consistent with prior single‐center and national studies. Additionally, and perhaps most importantly, our study did not capture patient goals—the content of patient narrative and goals that drive nuanced shared decision‐making—limiting our ability to assess the impact of patient values on the CD patterns we observed. These may be obtained through mixed‐method design and patient interviews. Further, our institution is in Tennessee, where the Human Life Protection Act restricts access to abortion care [6]. The impact of the legal context may influence decisions regarding mode of delivery, although the content of counseling conversations was unable to be captured retrospectively.

Our study has several strengths. First, starting from our participant selection, LLFD status was determined through manual chart review by a board‐certified MFM specialist, with ambiguous cases adjudicated jointly with a board‐certified neonatologist. This subspecialist‐level review—rather than algorithmic case identification or abstraction by non‐clinical personnel—ensured rigorous application of the ACOG definition to a diagnostically heterogeneous condition that lacks a standardized coding framework. Second, we restricted our cohort to six LLFDs, minimizing the heterogeneity of our cohort and ensuring that our findings apply to diagnoses within a range of clinical and prognostic ambiguity. Third, our study examined indications for CD rather than merely the mode of delivery itself, with the aim of understanding the reasons behind LLFD cesarean deliveries. This offers insight into the clinical reasoning that drives mode of delivery in this population.

## 5 CONCLUSION

In this retrospective cohort study, nearly half of patients with an LLFD underwent cesarean delivery, driven predominantly by fetal indications rather than maternal indications and with considerable variation across specific diagnoses. When neonatal survival is expected to be brief or unlikely, fetal‐centered CD exposes patients to surgical risk despite limited anticipated neonatal benefit, raising the concern that the observed predominance of fetal indications for CD may not serve maternal safety.

This pattern, however, is not inherently a marker of misaligned decision‐making. For some patients, they may be willing to accept surgical risk to maximize an opportunity for a live birth, with the goal of bonding or other experiences they find meaningful. Because this study did not assess patient goals, we cannot determine whether the observed elevation in CD rate ultimately aligns with such preferences, and further research centering patient goals in LLFD delivery planning is needed to further inform counseling frameworks for this population. Our findings underscore the importance of high‐quality shared decision‐making in establishing that the mode of delivery is responsive to patient goals while promoting maternal safety. Clinicians must avoid applying assumptions from uncomplicated pregnancies into their risk‐benefit assessments, yet recognize that CD could nonetheless be the best mode for achieving patient‐specified benefits of an LLFD pregnancy and delivery.

## AUTHOR CONTRIBUTIONS

**B. Wade Hamilton**: Writing—review and editing. **Luke A. Gatta**: Conceptualization; investigation; writing—review and editing; supervision; resources. **Alexandra C. Sundermann**: Conceptualization; methodology; visualization; writing—review and editing; formal analysis; software. **Halle C. Petrie**: Conceptualization; investigation; funding acquisition; writing—original draft; methodology; validation; visualization. **Emily A. Morris**: Conceptualization; writing—review and editing. **Allison M. McCarthy**: writing—review and editing; conceptualization.

## CONFLICT OF INTEREST STATEMENT

The authors declare no conflicts of interest.

## ETHICS STATEMENT

This study was approved by the Vanderbilt University Medical Center Institutional Review Board (IRB #250859). A waiver of informed consent was granted given the retrospective nature of the study.

## Data Availability

The data that support the findings of this study are available on request from the corresponding author. No material from previously published sources has been reproduced in this manuscript. All figures and tables are original work of the authors.
